# Supplementary material for: Association of Metabolites, Nutrients, and Toxins in Maternal and Cord Serum with Asthma, IgE, SPT, FeNO, and Lung Function in Offspring
Source: Metabolites. 2023 Jun 9;13(6):737. doi: 10.3390/metabo13060737 (PMC10301645; doi:10.3390/metabo13060737)
Supplement: Supplementary file 1 [file metabolites-13-00737-s001.zip › metabolites-2378849-supplementary.pdf]

Supplemental Materials

for the manuscript

Association of metabolites, nutrients, and toxins in maternal and cord serum with asthma, IgE, SPT, FeNO, and lung function in offspring

Wilfried Karmaus [1,*], Parnian Kheirkhah Rahimabad [1,†], Ngan Pham [1], Nandini Mukherjee [2,†], Su Chen [3], Thilani M. Anthony [4], Hasan S. Arshad [5,6], Aniruddha Rathod [7], Nahid Sultana [1] and A. Daniel Jones [4]

[1] Division of Epidemiology, Biostatistics, and Environmental Health, School of Public Health, University of Memphis, Memphis, TN 38152, USA; parnian.k@memphis.edu (P.K.R.); nahidkmc@gmail.com (N.S.); nganphamphuong@gmail.com (N.P.)
[2] Department of Epidemiology, Fay W. Boozman College of Public Health, University of Arkansas for Medical Sciences, Little Rock, AR 72205, USA; nmukherjee@uams.edu
[3] Department of Biostatistics, College of Public Health, University of Nebraska Medical Center, Omaha, NE 68198-4375, USA; suchen@unmc.edu
[4] Department of Biochemistry & Molecular Biology, Michigan State University, East Lansing, MI 48824, USA; thilani@chemistry.msu.edu (T.M.A.); jonesar4@msu.edu (A.D.J.)
[5] Clinical and Experimental Sciences, Faculty of Medicine, University of Southampton, Southampton SO17 1BJ, UK; s.h.arshad@soton.ac.uk
[6] David Hide Asthma and Allergy Research Centre, Isle of Wight PO30 5TG, UK
[7] Peter O'Donnell Jr. School of Public Health, University of Texas Southwestern Medical Center, Dallas, TX 75390, USA; aniruddhabhadresh.rathod@utsouthwestern.edu
* Correspondence: karmaus1@memphis.edu
† These authors contributed equally to this work.

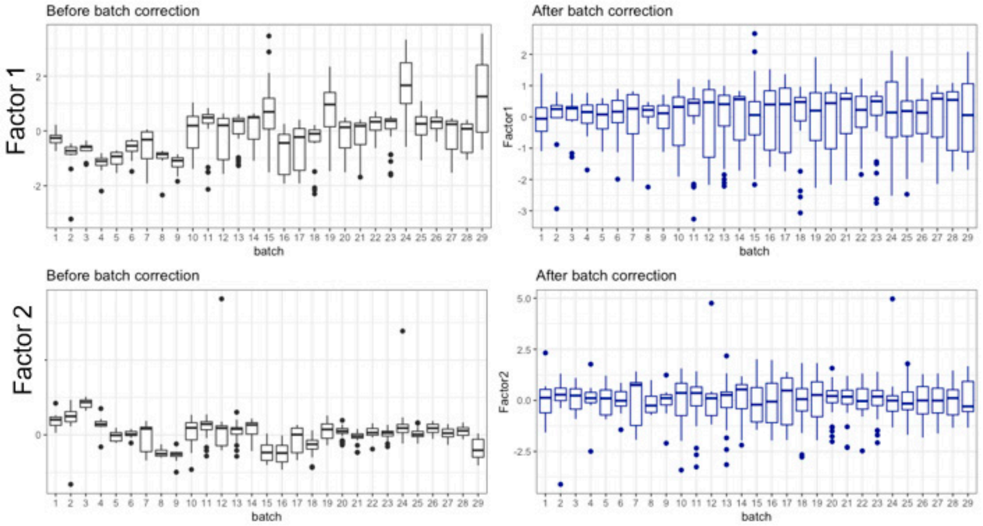

Figure S1: Principal component analyses of four internal standards for non-rank-transformed continuous polar MNTs before and after batch corrections. For polar MNTs, we measured signals of four internal standards: cotinine-d_3 [1.22_180.1208m/z], [$^{13}C_3$]caffeine [1.05_215.1008n], valine-d_8 [6.94_125.1291n], and phenylalanine-d_5 [5.71_170.1102n] and identified two principal components, which were related to batches. To mark these, two dummy variables were used (dummy 1: batches 19, 24, 29, and 15; dummy 2: batches 1-9), which removes the batch effects.

Processing of LC/HRMS data

Polar MNTs

Peak alignment, detection, normalization, and annotation were performed using Progenesis QI v2.4 software (Waters, Nonlinear Dynamics, Newcastle upon Tyne NE1 2JE, UK). Annotations were suggested by searching spectra extracted using Compound Discoverer software (Thermo) against the mzCloud database (Thermo), followed by manual searching of Human Metabolome Database, Metabolomics Workbench, and METLIN, with additional manual verification of characteristic fragment ions presence in the high collision-energy mass spectra. Peak areas were exported from Progenesis software and filtered in Microsoft Excel to remove signals with highest abundances in blanks and those with relative mass defect (RMD) > 1200 ppm, as these are often ascribed to inorganic salts. Exported peak areas were normalized to the area of the internal standard cotinine-d_3 and scaled by multiplication by 1×10^4.

Non-polar MNTs

Peak alignment, detection, normalization, and annotation were performed using Progenesis QI v2.4 software (Waters, Nonlinear Dynamics, Newcastle upon Tyne NE1 2JE, UK). MNT annotations were performed separately for lipid (MS^E spectra) based on several metrics: (1) agreement of ion masses reported by Progenesis QI software for aggregate spectra for the entire data set must agree to within 10 ppm of theoretical values; (2) results of searching high-energy mass spectra against HMDB, LipidBlast, LipidMaps, Metabolomics Workbench, and ChemSpider libraries using 10 ppm mass error window; (3) manual matching of fragment ions in high-energy mass spectra with MassBank of North America MS/MS spectrum database; and (4) manual interpretation of high collision energy mass spectra (particularly oxidized lipids). For negative-ion lipid data, filtering removed all signals highest in blank samples and removed all signals with RMD > 1200 ppm. Signals were normalized to the combined signals from the six MNTs of the highest maximum abundance signals then these values were multiplied by 1×10^6. For neutral lipids detected using positive-ion mode flow injection analysis (FIA), peak alignment, detection, normalization, and annotation were performed using Progenesis QI v. 3.0 (Waters) software using Direct Sample Analysis for FIA data. Positive-ion data for neutral lipids were normalized to the total signal, followed by filtering to remove phosphatidylcholine and sphingomyelin lipids that were previously reported in the negative-ion lipid data. The resulting signals for each lipid were then multiplied by 10^3.

Sample preparation for analysis of metabolites, nutrients, and toxins

Sera were grouped, processed, and analyzed in random order, with each batch including analyses of multiple blanks, pooled quality control extracts, and extracts of reference serum (Sigma). Polypropylene microcentrifuge tubes were loaded with 25 μL aliquots each of water-soluble and

organic-soluble stable isotope-labeled internal standard cocktails [Internal Standard Solution #A1: 5.0 μM succinic acid-d_4, 1.0 μM cotinine-d_3, 5.0 μM L-valine-d_8, 5.0 μM [$^{13}C_3$]caffeine, 5.0 μM thymine-d_3, and 5.0 μM phenylalanine-d_5 in acetonitrile/water (90:10 v/v); Internal Standard Solution #L1: 1 μM palmitic acid-d_{31}, 2 μM 1,2-dimyristoyl-d_{54}-sn-glycero-3-phosphocholine, and 1 μM [β-estradiol-16,16,17-d_3 in acetonitrile]. An additional 150 μL of acetonitrile was added to each tube, followed by 20 μL of blood serum after thawing on ice. Each tube was vortexed and held on ice. To each tube was added 200 μL of MTBE (methyl *tert*-butyl ether) and 400 μL of MilliQ water. Tubes were vortexed and centrifuged (10,000 x g, 4˚C, 15 min). A pipette was used to collect 180 μL from the top organic layer, and another 200-μL volume of MTBE was added to the aqueous (lower) layer. After vortexing and centrifuging (as above), 180 μL of the upper layer was combined with the first MTBE fraction, and this "nonpolar" fraction was evaporated to dryness under a stream of nitrogen gas, then stored at -20˚C. The lower (polar) fraction was evaporated to dryness under vacuum using a SpeedVac without application of heat. Prior to analysis, non-polar fractions were dissolved in 1 mL of 2-propanol/water (90:10 v/v) and polar fractions in 200 μL of acetonitrile/water (90:10 v/v), and aliquots transferred to glass autosampler vials.

Profiling of Metabolites

Profiling of polar fraction metabolites was performed using a Thermo QExactive mass spectrometer interfaced to a Thermo Vanquish Binary Flex pump and autosampler. Polar metabolites were analyzed using positive-ion mode electrospray ionization. Chromatographic separations were performed using a Waters Acquity BEH Amide column (10 cm X 1.0 mm, 1.7 μm) held at 30˚C. Mass spectra were acquired using All-Ions Fragmentation over *m/z* 70-1050 at mass resolution (at *m/z* 200) of 70000 (full scan) and 35000 (AIF) with automatic gain control (AGC) target of 3×10^6 and stepped collision energy of 10-30-60 normalized collision energy (NCE). Mobile phases were solvent A: 100 mM ammonium acetate in water: acetonitrile (50: 50 v/v adjusted to pH 9.0 with 0.4% ammonium hydroxide before mixing) and solvent B: 10 mM ammonium acetate in water: acetonitrile (10: 90 v/v with 0.04% ammonium hydroxide, pH 9.0) using a flow rate of 0.3 mL/min with a gradient elution as follows: 99% B from 0–1 min, followed by a linear increase to 50% B at 7.0 min, then a hold at 50% B until 10.0 min. Finally, the solvent returned to the initial conditions (99% B) at 10.01 min and equilibrated for 5 more mins (15 min total run time). Peak alignment, detection, normalization, and annotation were performed using Progenesis QI v2.4 software (Waters), with annotations suggested by searching spectra extracted using Compound Discoverer software (Thermo) against the mzCloud database (Thermo), followed by manual verification of the presence of characteristic fragment ions in the high collision-energy mass spectra. Peak areas exported from Progenesis QI software were filtered to remove signals with highest abundances in blanks and those with relative mass defect (RMD) > 1200 ppm, [60, 61] as these are often attributable to inorganic salts. Ions attributed to salt clusters eluting at 6.5 and 7.2 minutes were also removed. Exported peak areas were normalized to the area for the internal standard cotinine-d_3, then scaled by multiplication by 1×10^4. Conversions to μg/L were based on empirical determinations of relative response factors when authentic standards were commercially available, and values for the most chemically similar metabolites were used when standards were not available. Annotations were verified by matching

chromatographic retention times and masses to authentic standards (Cayman Chemicals, Ann Arbor, MI USA).

Nonpolar fraction analysis

Redissolved MTBE fractions were analyzed using reversed-phase LC/MS^E and negative-ion mode electrospray ionization. Separations employed a Supelco Ascentis Express C18 column (10 cm x 2.1 mm, 2.7 μm) held at 50 °C, and mobile phases A: 10 mM ammonium formate in water: acetonitrile (80: 20 v/v) and B: 1 mM ammonium formate in isopropanol: acetonitrile (90: 10 v/v). Total flow rate was 0.3 mL/min with linear gradient elution as follows: hold at 0% B from 0–1 min, increase to 40% B at 3.0 min, 43% B at 5.0 min, 50% B at 5.50 min, 54% B at 12.00 min, 70% B at 13 min, 99% B at 22 min, followed by a hold at 99% B until 28 min. At 28.01 min, the solvent returned to the initial conditions and was held at 0% B until 30 min. For each analysis, a 10 μL volume of each extract was injected.

Replication p-values

MNTs that fulfilled an FDR-adjusted p-value of 0.05 were tested for replication in the F2-generation. In F2, we used a p-value cut-off of 0.2 for replication. Since a null hypothesis of "H0: no association between an MNT and health outcome" will be rejected only when tests in both F1 and F2 generation data are statistically significant, the actual Type I error for the two tests together should be P(Reject H0 in F1 and Reject H0 in F2 data given H0 is true)=P(Reject H0 in F1 data given H0 is true)*P(Reject H0 in F2 data given H0 is true) if assuming F1 and F2 are independent. Using a cut-off FDR adjusted p-value $<\sqrt{0.05} \approx 0.2$ in the discovery analyses and a raw p-value $<\sqrt{0.05} \approx 0.2$, the empirical FDR for the discovery and replication analyses together is close to 0.05 (manuscripts in progress). In this study, to be on the conservative side, we used an FDR adjusted p-value<0.05 in the discovery analyses (F1), but raw p-value <0.2 in the replication analyses (F2).empirical FDR for the discovery and replication analyses together is close to 0.05 (manuscripts in progress).

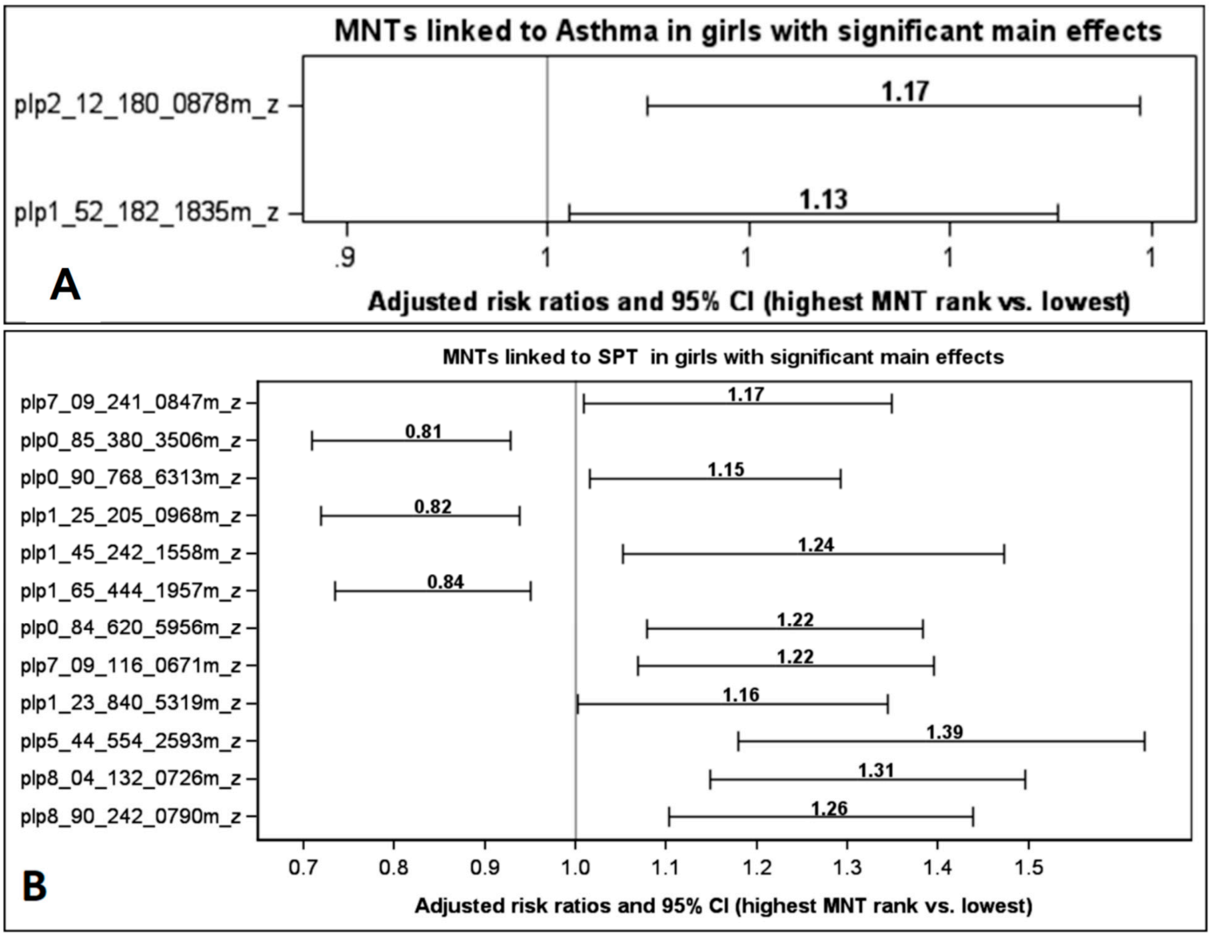

MNTs linked to Asthma in girls with significant main effects
plp2_12_180_0878m_z
plp1_52_182_1835m_z
1.17
1.13
.9
1
1
1
1
A
Adjusted risk ratios and 95% CI (highest MNT rank vs. lowest)
MNTs linked to SPT in girls with significant main effects
plp7_09_241_0847m_z
plp0_85_380_3506m_z
plp0_90_768_6313m_z
plp1_25_205_0968m_z
plp1_45_242_1558m_z
plp1_65_444_1957m_z
plp0_84_620_5956m_z
plp7_09_116_0671m_z
plp1_23_840_5319m_z
plp5_44_554_2593m_z
plp8_04_132_0726m_z
plp8_90_242_0790m_z
1.17
0.81
1.15
0.82
1.24
0.84
1.22
1.22
1.16
1.39
1.31
1.26
0.7
0.8
0.9
1.0
1.1
1.2
1.3
1.4
1.5
B
Adjusted risk ratios and 95% CI (highest MNT rank vs. lowest)

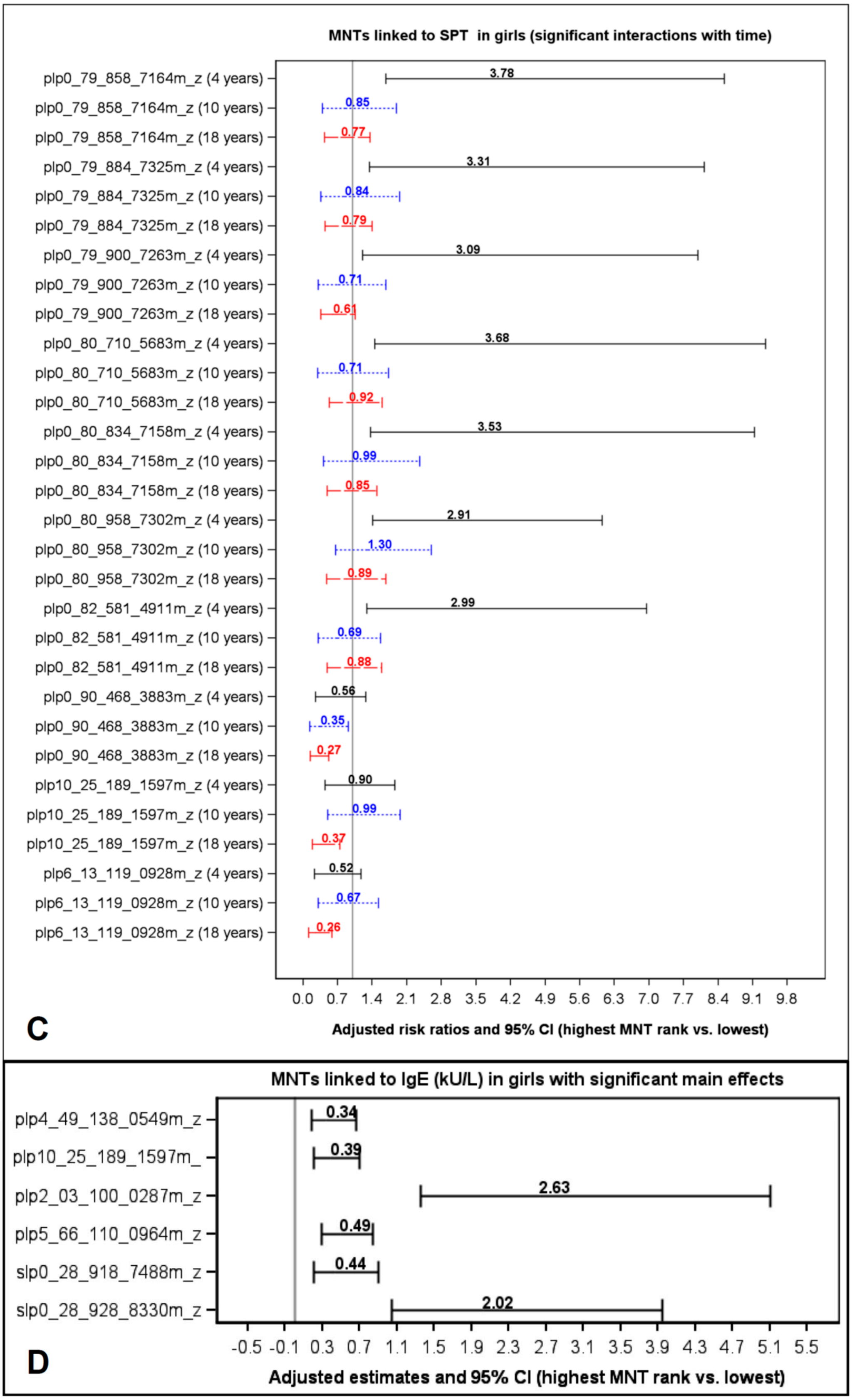

MNTs linked to SPT in girls (significant interactions with time)
plp0_79_858_7164m_z (4 years)
plp0_79_858_7164m_z (10 years)
plp0_79_858_7164m_z (18 years)
plp0_79_884_7325m_z (4 years)
plp0_79_884_7325m_z (10 years)
plp0_79_884_7325m_z (18 years)
plp0_79_900_7263m_z (4 years)
plp0_79_900_7263m_z (10 years)
plp0_79_900_7263m_z (18 years)
plp0_80_710_5683m_z (4 years)
plp0_80_710_5683m_z (10 years)
plp0_80_710_5683m_z (18 years)
plp0_80_834_7158m_z (4 years)
plp0_80_834_7158m_z (10 years)
plp0_80_834_7158m_z (18 years)
plp0_80_958_7302m_z (4 years)
plp0_80_958_7302m_z (10 years)
plp0_80_958_7302m_z (18 years)
plp0_82_581_4911m_z (4 years)
plp0_82_581_4911m_z (10 years)
plp0_82_581_4911m_z (18 years)
plp0_90_468_3883m_z (4 years)
plp0_90_468_3883m_z (10 years)
plp0_90_468_3883m_z (18 years)
plp10_25_189_1597m_z (4 years)
plp10_25_189_1597m_z (10 years)
plp10_25_189_1597m_z (18 years)
plp6_13_119_0928m_z (4 years)
plp6_13_119_0928m_z (10 years)
plp6_13_119_0928m_z (18 years)
3.78
0.85
0.77
3.31
0.84
0.79
3.09
0.71
0.61
3.68
0.71
0.92
3.53
0.99
0.85
2.91
1.30
0.89
2.99
0.69
0.88
0.56
0.35
0.27
0.90
0.99
0.37
0.52
0.67
0.26
0.0 0.7 1.4 2.1 2.8 3.5 4.2 4.9 5.6 6.3 7.0 7.7 8.4 9.1 9.8
Adjusted risk ratios and 95% CI (highest MNT rank vs. lowest)
C
MNTs linked to IgE (kU/L) in girls with significant main effects
plp4_49_138_0549m_z
plp10_25_189_1597m_
plp2_03_100_0287m_z
plp5_66_110_0964m_z
slp0_28_918_7488m_z
slp0_28_928_8330m_z
0.34
0.39
2.63
0.49
0.44
2.02
-0.5 -0.1 0.3 0.7 1.1 1.5 1.9 2.3 2.7 3.1 3.5 3.9 4.3 4.7 5.1 5.5
Adjusted estimates and 95% CI (highest MNT rank vs. lowest)
D

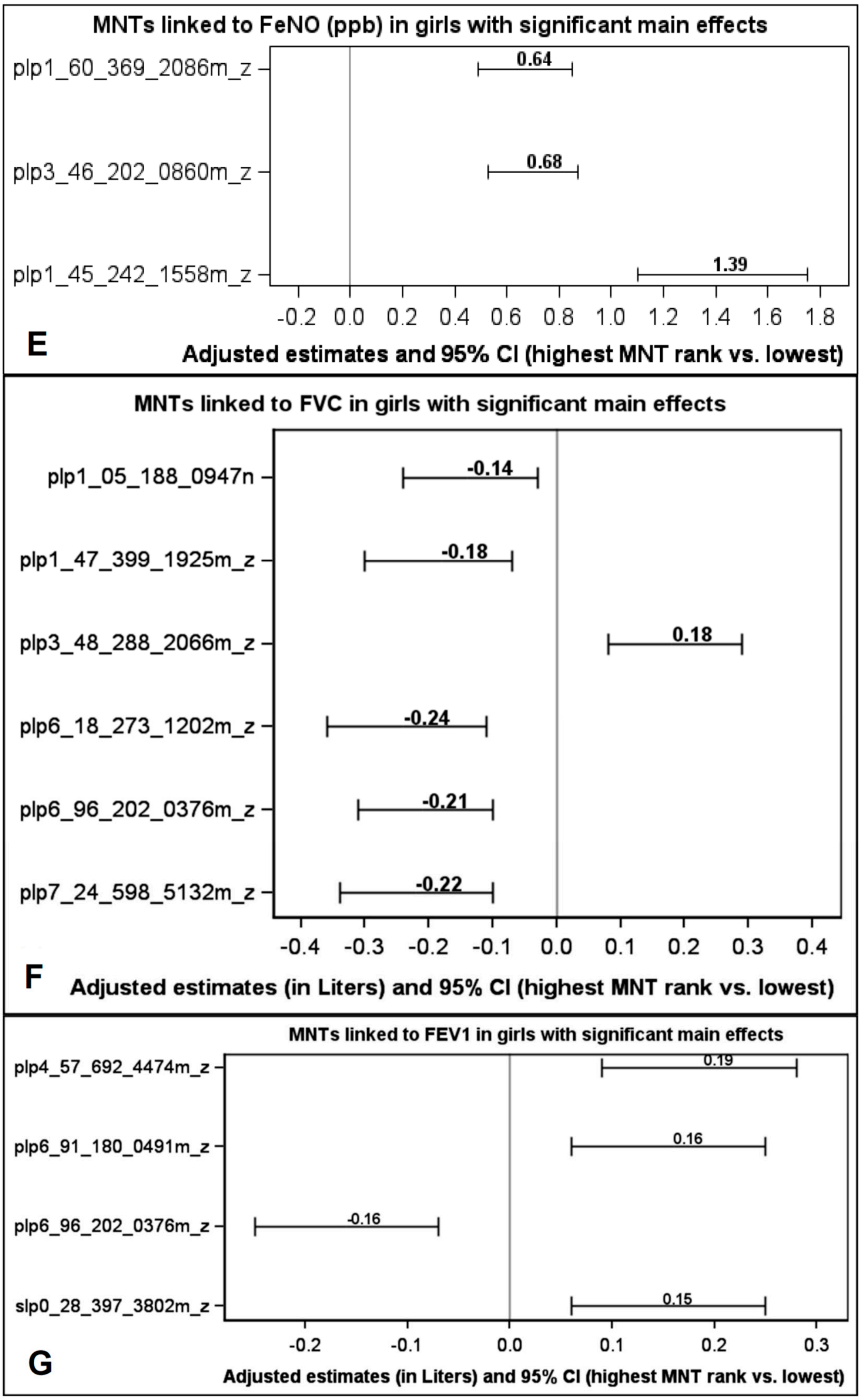

MNTs linked to FeNO (ppb) in girls with significant main effects
plp1_60_369_2086m_z
0.64
plp3_46_202_0860m_z
0.68
plp1_45_242_1558m_z
1.39
-0.2 0.0 0.2 0.4 0.6 0.8 1.0 1.2 1.4 1.6 1.8
E
Adjusted estimates and 95% CI (highest MNT rank vs. lowest)
MNTs linked to FVC in girls with significant main effects
plp1_05_188_0947n
-0.14
plp1_47_399_1925m_z
-0.18
plp3_48_288_2066m_z
0.18
plp6_18_273_1202m_z
-0.24
plp6_96_202_0376m_z
-0.21
plp7_24_598_5132m_z
-0.22
-0.4 -0.3 -0.2 -0.1 0.0 0.1 0.2 0.3 0.4
F
Adjusted estimates (in Liters) and 95% CI (highest MNT rank vs. lowest)
MNTs linked to FEV1 in girls with significant main effects
plp4_57_692_4474m_z
0.19
plp6_91_180_0491m_z
0.16
plp6_96_202_0376m_z
-0.16
slp0_28_397_3802m_z
0.15
-0.2 -0.1 0.0 0.1 0.2 0.3
G
Adjusted estimates (in Liters) and 95% CI (highest MNT rank vs. lowest)

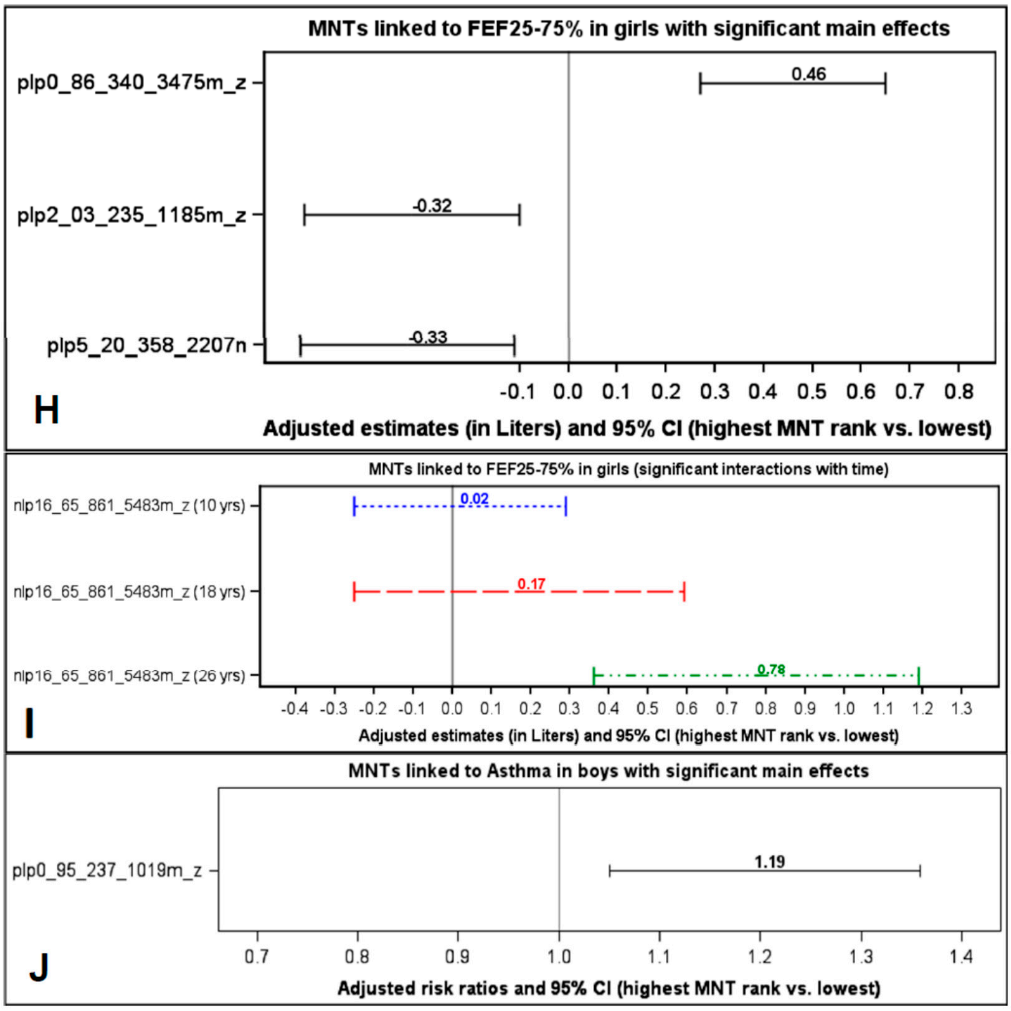
MNTs linked to FEF25-75% in girls with significant main effects
plp0_86_340_3475m_z
plp2_03_235_1185m_z
plp5_20_358_2207n
0.46
-0.32
-0.33
-0.1 0.0 0.1 0.2 0.3 0.4 0.5 0.6 0.7 0.8
H
Adjusted estimates (in Liters) and 95% CI (highest MNT rank vs. lowest)
MNTs linked to FEF25-75% in girls (significant interactions with time)
nlp16_65_861_5483m_z (10 yrs)
nlp16_65_861_5483m_z (18 yrs)
nlp16_65_861_5483m_z (26 yrs)
0.02
0.17
0.78
-0.4 -0.3 -0.2 -0.1 0.0 0.1 0.2 0.3 0.4 0.5 0.6 0.7 0.8 0.9 1.0 1.1 1.2 1.3
I
Adjusted estimates (in Liters) and 95% CI (highest MNT rank vs. lowest)
MNTs linked to Asthma in boys with significant main effects
plp0_95_237_1019m_z
1.19
0.7 0.8 0.9 1.0 1.1 1.2 1.3 1.4
J
Adjusted risk ratios and 95% CI (highest MNT rank vs. lowest)

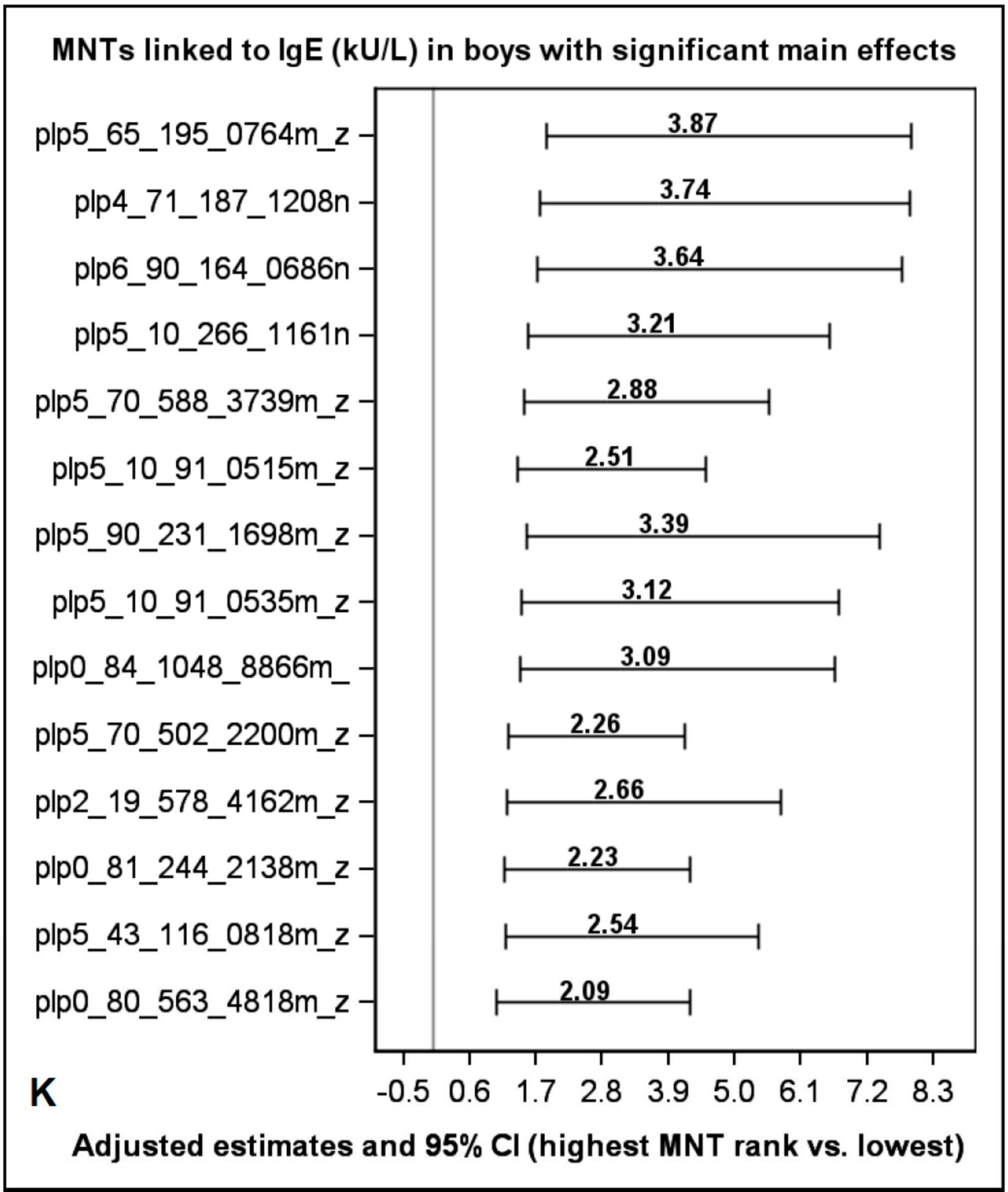
MNTs linked to IgE (kU/L) in boys with significant main effects
plp5_65_195_0764m_z
plp4_71_187_1208n
plp6_90_164_0686n
plp5_10_266_1161n
plp5_70_588_3739m_z
plp5_10_91_0515m_z
plp5_90_231_1698m_z
plp5_10_91_0535m_z
plp0_84_1048_8866m_
plp5_70_502_2200m_z
plp2_19_578_4162m_z
plp0_81_244_2138m_z
plp5_43_116_0818m_z
plp0_80_563_4818m_z
3.87
3.74
3.64
3.21
2.88
2.51
3.39
3.12
3.09
2.26
2.66
2.23
2.54
2.09
-0.5 0.6 1.7 2.8 3.9 5.0 6.1 7.2 8.3
K
Adjusted estimates and 95% CI (highest MNT rank vs. lowest)

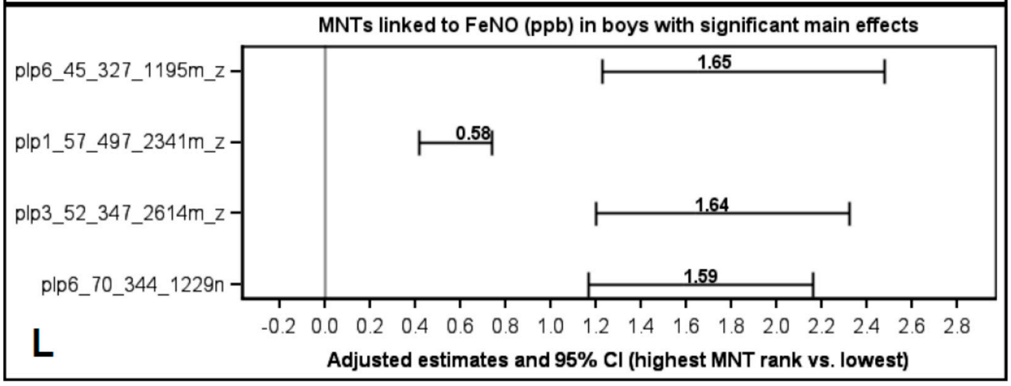
MNTs linked to FeNO (ppb) in boys with significant main effects
plp6_45_327_1195m_z
plp1_57_497_2341m_z
plp3_52_347_2614m_z
plp6_70_344_1229n
1.65
0.58
1.64
1.59
-0.2 0.0 0.2 0.4 0.6 0.8 1.0 1.2 1.4 1.6 1.8 2.0 2.2 2.4 2.6 2.8
L
Adjusted estimates and 95% CI (highest MNT rank vs. lowest)

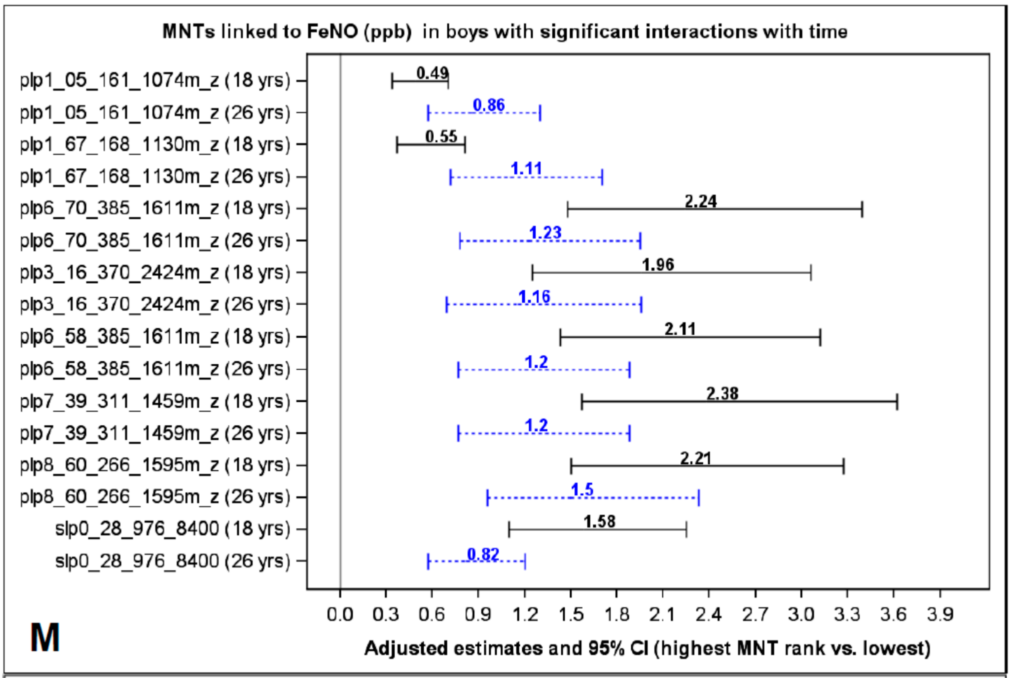
MNTs linked to FeNO (ppb) in boys with significant interactions with time
plp1_05_161_1074m_z (18 yrs)
plp1_05_161_1074m_z (26 yrs)
plp1_67_168_1130m_z (18 yrs)
plp1_67_168_1130m_z (26 yrs)
plp6_70_385_1611m_z (18 yrs)
plp6_70_385_1611m_z (26 yrs)
plp3_16_370_2424m_z (18 yrs)
plp3_16_370_2424m_z (26 yrs)
plp6_58_385_1611m_z (18 yrs)
plp6_58_385_1611m_z (26 yrs)
plp7_39_311_1459m_z (18 yrs)
plp7_39_311_1459m_z (26 yrs)
plp8_60_266_1595m_z (18 yrs)
plp8_60_266_1595m_z (26 yrs)
slp0_28_976_8400 (18 yrs)
slp0_28_976_8400 (26 yrs)
0.49
0.86
0.55
1.11
2.24
1.23
1.96
1.16
2.11
1.2
2.38
1.2
2.21
1.5
1.58
0.82
0.0 0.3 0.6 0.9 1.2 1.5 1.8 2.1 2.4 2.7 3.0 3.3 3.6 3.9
Adjusted estimates and 95% CI (highest MNT rank vs. lowest)
M

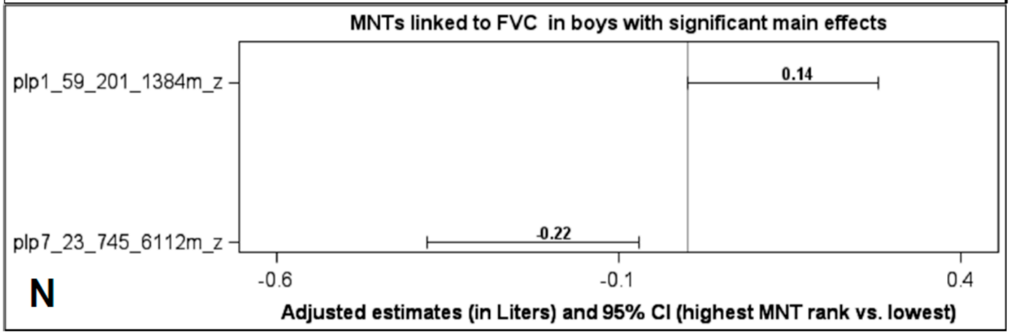
MNTs linked to FVC in boys with significant main effects
plp1_59_201_1384m_z
plp7_23_745_6112m_z
0.14
-0.22
-0.6
-0.1
0.4
Adjusted estimates (in Liters) and 95% CI (highest MNT rank vs. lowest)
N

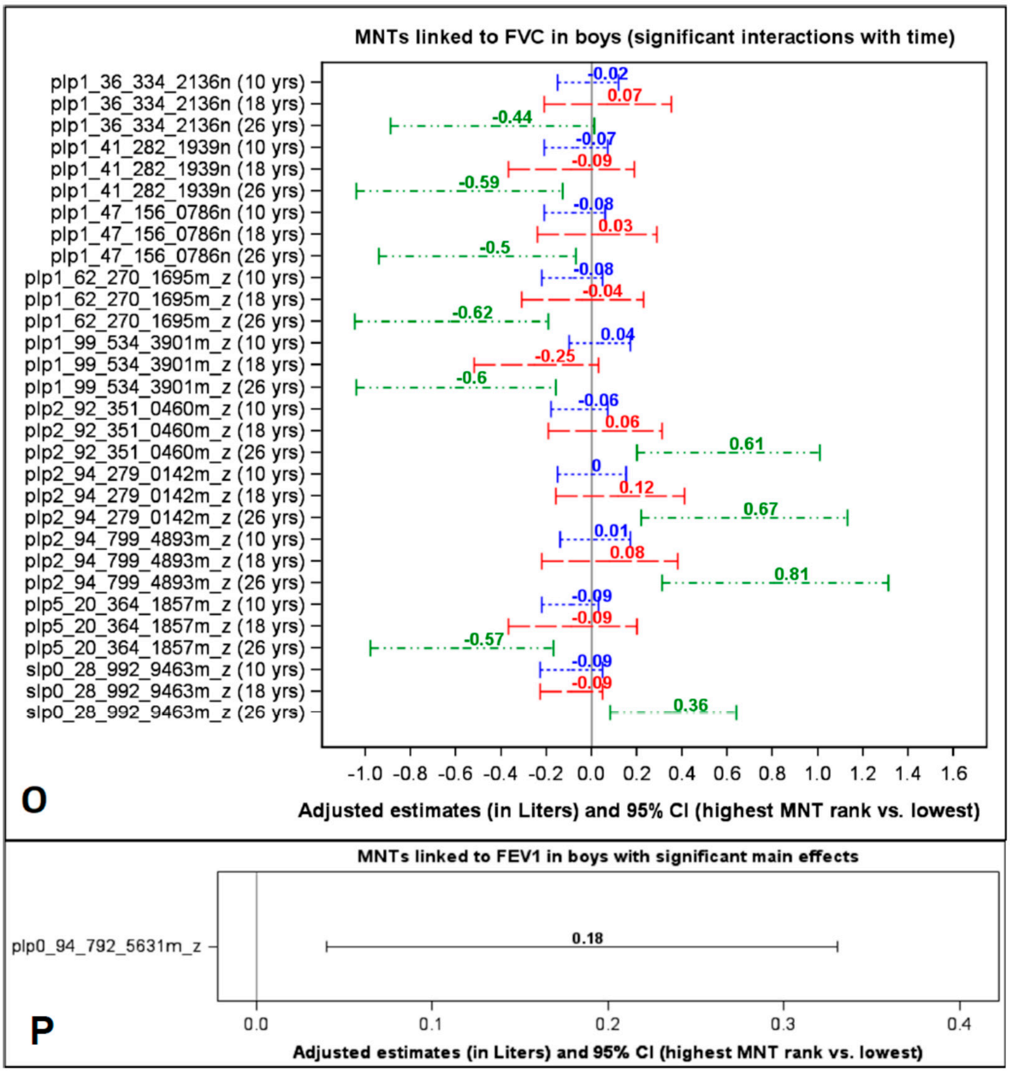

MNTs linked to FVC in boys (significant interactions with time)
plp1_36_334_2136n (10 yrs)
plp1_36_334_2136n (18 yrs)
plp1_36_334_2136n (26 yrs)
plp1_41_282_1939n (10 yrs)
plp1_41_282_1939n (18 yrs)
plp1_41_282_1939n (26 yrs)
plp1_47_156_0786n (10 yrs)
plp1_47_156_0786n (18 yrs)
plp1_47_156_0786n (26 yrs)
plp1_62_270_1695m_z (10 yrs)
plp1_62_270_1695m_z (18 yrs)
plp1_62_270_1695m_z (26 yrs)
plp1_99_534_3901m_z (10 yrs)
plp1_99_534_3901m_z (18 yrs)
plp1_99_534_3901m_z (26 yrs)
plp2_92_351_0460m_z (10 yrs)
plp2_92_351_0460m_z (18 yrs)
plp2_92_351_0460m_z (26 yrs)
plp2_94_279_0142m_z (10 yrs)
plp2_94_279_0142m_z (18 yrs)
plp2_94_279_0142m_z (26 yrs)
plp2_94_799_4893m_z (10 yrs)
plp2_94_799_4893m_z (18 yrs)
plp2_94_799_4893m_z (26 yrs)
plp5_20_364_1857m_z (10 yrs)
plp5_20_364_1857m_z (18 yrs)
plp5_20_364_1857m_z (26 yrs)
slp0_28_992_9463m_z (10 yrs)
slp0_28_992_9463m_z (18 yrs)
slp0_28_992_9463m_z (26 yrs)
-0.02
0.07
-0.44
-0.07
-0.09
-0.59
-0.08
0.03
-0.5
-0.08
-0.04
-0.62
0.04
-0.25
-0.6
-0.06
0.06
0.61
0
0.12
0.67
0.01
0.08
0.81
-0.09
-0.09
-0.57
-0.09
-0.09
0.36
-1.0 -0.8 -0.6 -0.4 -0.2 0.0 0.2 0.4 0.6 0.8 1.0 1.2 1.4 1.6
O
Adjusted estimates (in Liters) and 95% CI (highest MNT rank vs. lowest)
MNTs linked to FEV1 in boys with significant main effects
plp0_94_792_5631m_z
0.18
0.0 0.1 0.2 0.3 0.4
P
Adjusted estimates (in Liters) and 95% CI (highest MNT rank vs. lowest)

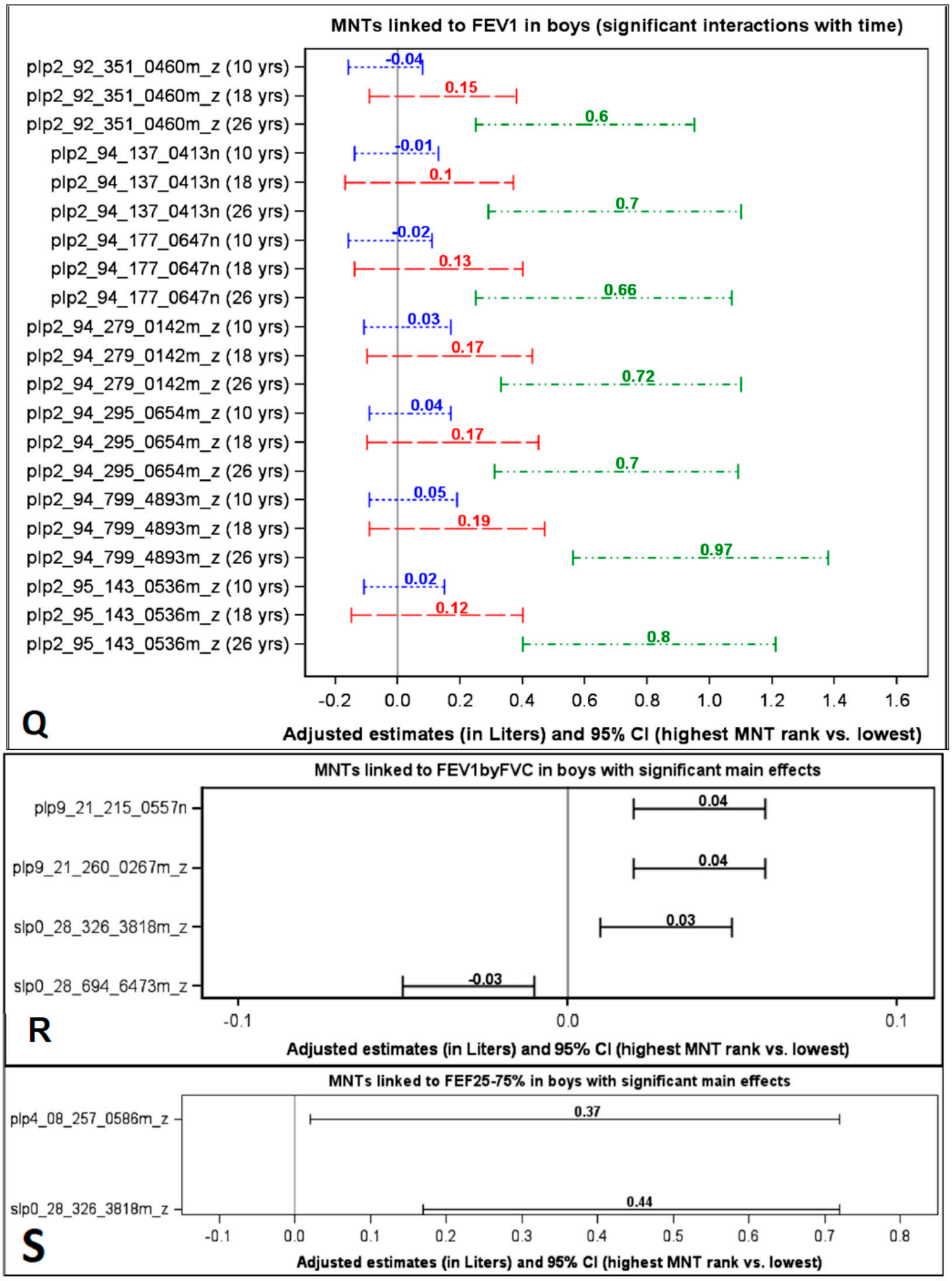

Figure S2.A.-S2.S. Associations of MNTs with different respiratory and allergic outcomes comparing the highest with the lowest quintile of MNTs.

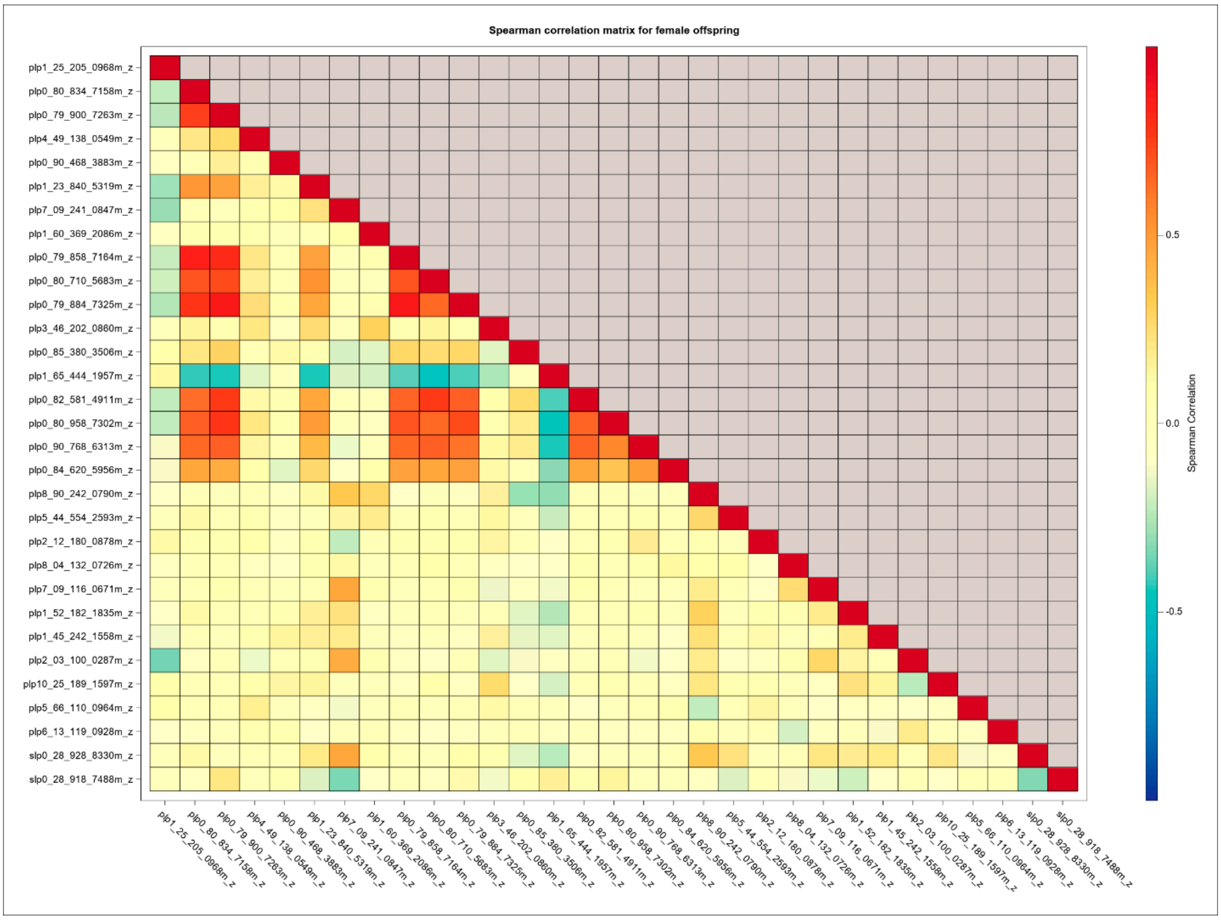

Figure S3.A. Heatmap of different metabolites, nutrients, toxins (MNTs) with multiple allergic and respiratory outcomes in female participants.

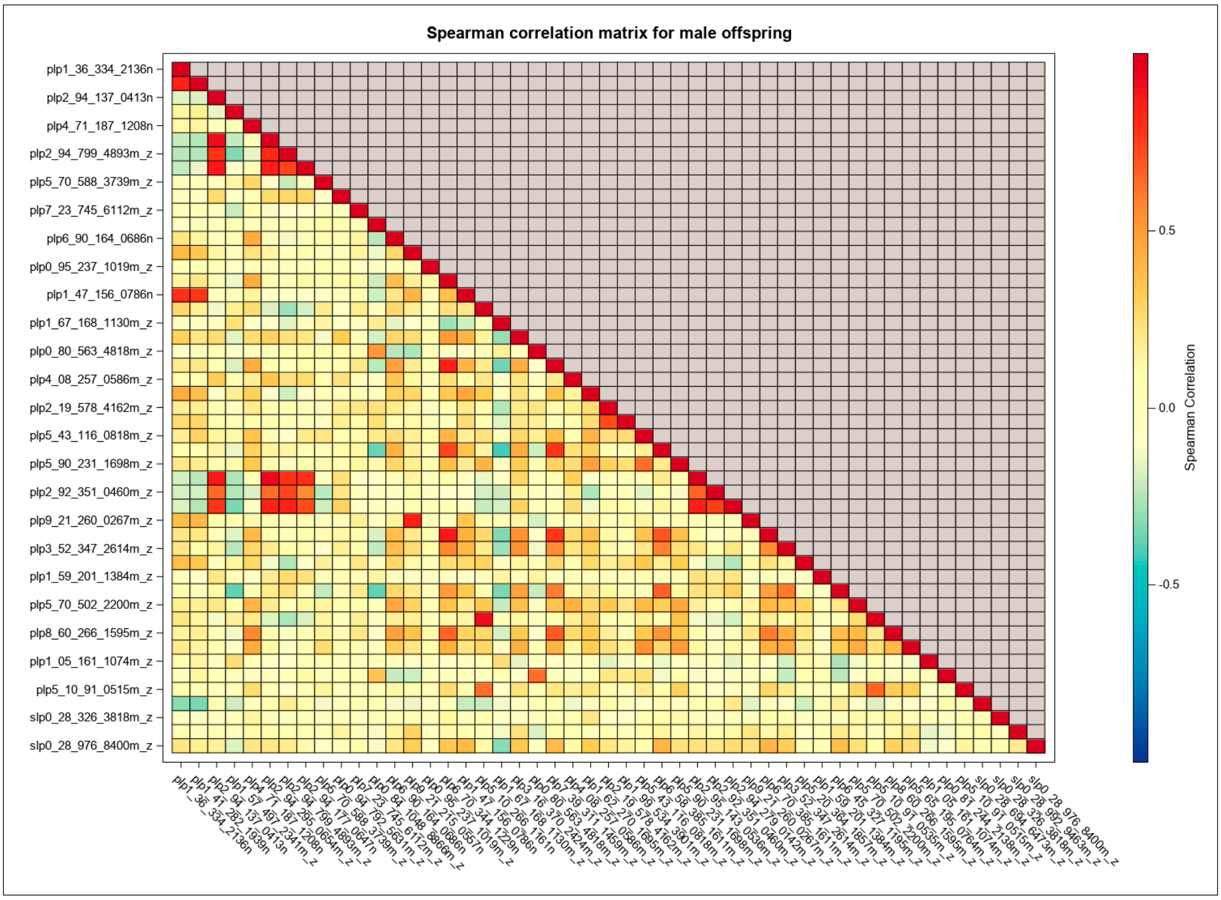

Figure S3.B. Heatmap of different metabolites, nutrients, toxins (MNTs) with multiple allergic and respiratory outcomes in male participants.

Table S1.A. MNTs with a Spearman Correlation coefficients >0.70 among female F1 participants (see Figure S2.B.)

MNT	MNT	Spearman correlation
plp0_79_858_7164m_z	plp0_80_710_5683m_z	0.70203
plp0_79_858_7164m_z	plp0_79_900_7263m_z	0.81267
plp0_79_858_7164m_z	plp0_80_834_7158m_z	0.85371
plp0_79_858_7164m_z	plp0_79_884_7325m_z	0.88199
plp0_79_884_7325m_z	plp0_80_958_7302m_z	0.71884
plp0_79_884_7325m_z	plp0_80_834_7158m_z	0.78467
plp0_79_884_7325m_z	plp0_79_900_7263m_z	0.87715
plp0_79_900_7263m_z	plp0_80_710_5683m_z	0.72118
plp0_79_900_7263m_z	plp0_80_834_7158m_z	0.75372
plp0_79_900_7263m_z	plp0_82_581_4911m_z	0.76965
plp0_79_900_7263m_z	plp0_80_958_7302m_z	0.77054
plp0_80_710_5683m_z	plp0_82_581_4911m_z	0.77465

Abbreviations: plp = polar MNT

Table S1.B. MNTs with Spearman correlation coefficients >0.70 among male F1 participants (see Figure S2.B.)

MNT	MNT	Spearman correlation
plp1_36_334_2136n	plp1_47_156_0786n	0.80045
plp1_36_334_2136n	plp1_41_282_1939n	0.83107
plp1_41_282_1939n	plp1_47_156_0786n	0.78768
plp1_99_534_3901m_z	plp2_19_578_4162m	0.72241
plp2_92_351_0460m_z	plp2_94_799_4893m	0.75615
plp2_92_351_0460m_z	plp2_94_279_0142m	0.76581
plp2_94_137_0413n	plp2_94_799_4893m	0.78737
plp2_94_137_0413n	plp2_94_279_0142m	0.79661
plp2_94_137_0413n	plp2_94_177_0647n	0.87513
plp2_94_137_0413n	plp2_95_143_0536m	0.88855
plp2_94_137_0413n	plp2_94_295_0654m	0.91439
plp2_94_177_0647n	plp2_94_279_0142m	0.73261
plp2_94_177_0647n	plp2_94_799_4893m	0.75163
plp2_94_177_0647n	plp2_95_143_0536m	0.81596
plp2_94_177_0647n	plp2_94_295_0654m	0.85852
plp2_94_279_0142m_z	plp2_94_295_0654m	0.84692
plp2_94_279_0142m_z	plp2_94_799_4893m	0.84817
plp2_94_279_0142m_z	plp2_95_143_0536m	0.86209
plp2_94_295_0654m_z	plp2_94_799_4893m	0.81871
plp2_94_295_0654m_z	plp2_95_143_0536m	0.93097
plp2_94_799_4893m_z	plp2_95_143_0536m	0.80993
plp5_10_266_1161n	plp5_10_91_0535m_	0.92661
plp6_58_385_1611m_z	plp6_70_385_1611m	0.70158
plp6_58_385_1611m_z	plp6_70_344_1229n	0.74648
plp6_58_385_1611m_z	plp7_39_311_1459m	0.78174
plp6_70_344_1229n	plp7_39_311_1459m	0.86227
plp6_70_344_1229n	plp6_70_385_1611m	0.88019
plp6_70_385_1611m_z	plp7_39_311_1459m	0.78027
plp9_21_215_0557n	plp9_21_260_0267m_z	0.8687

Abbreviations: plp = polar MNT
